# WE-KAN: SAR Image Rotated Object Detection Method Based on Wavelet Domain Feature Enhancement and KAN Prediction Head

**DOI:** 10.3390/s26072011

**Published:** 2026-03-24

**Authors:** Mingchun Li, Yang Liu, Qiang Wang, Dali Chen

**Affiliations:** 1 School of Intelligent Science and Information Engineering, Shenyang University, Shenyang 110044, China; wangqiang@syu.edu.cn; 2 College of Information Science and Engineering, Northeastern University, Shenyang 110819, China; chendali@ise.neu.edu.cn

**Keywords:** synthetic aperture radar (SAR), rotated object detection, wavelet transform, Kolmogorov–Arnold network (KAN)

## Abstract

Synthetic aperture radar (SAR) imagery plays a vital role in critical applications such as military reconnaissance and disaster monitoring. These applications require high detection accuracy. Therefore, rotated object detection has gained increasing attention. By predicting an object orientation angle, it offers advantages over horizontal bounding boxes, especially for elongated structures such as ships and bridges in SAR scenes. However, challenges such as speckle noise and complex backgrounds in SAR imagery still hinder high-precision detection. To address this, we propose WE-KAN, a novel rotated object detection framework based on wavelet features and Kolmogorov–Arnold network (KAN) prediction. First, we enhance the backbone by incorporating wavelet domain features from SAR grayscale images. The extracted wavelet domain features and image features are fused by a proposed attention module. Second, considering the sensitivity to angle prediction, we design a angle predictor based on KAN. This architecture provides a powerful and dedicated solution for accurate angle regression. Finally, for precise rotated bounding box regression, we employ a joint loss function combining a rotated intersection over union (RIoU) with a Gaussian distance loss function. These designs improve the model’s robustness to noise and its perception of fine object structures. When evaluated on the large-scale public RSAR dataset, our method achieves an AP50 of 70.1 and a mAP of 35.9 under the same training schedule and backbone network, significantly outperforming existing baselines. This demonstrates the effectiveness and robustness of our method for dense, small, and highly oriented objects in complex SAR scenes.

## 1. Introduction

Synthetic aperture radar is an advanced active microwave remote sensing imaging system [1]. It uses a radar antenna mounted on a satellite or aircraft platform. While in motion, it continuously transmits microwave pulses toward the ground and receives their echoes. This achieves multi-angle continuous observation of the same ground area [2]. The collected signals are synthesized into high-resolution images through coherent imaging algorithms. These images can depict the geometric and scattering characteristics of the Earth’s surface. This meets the demand for high-precision detection. Unlike natural optical images, SAR has unique advantages. Optical images rely on lighting conditions and are susceptible to weather and day-night variations. In contrast, SAR enables all-weather and all-day imaging [3]. As such, it holds broad and important application value in fields such as military reconnaissance [4], ocean studies [5], and disaster assessment [6].

With the advancement of computer vision technology [7,8] and the availability of more public datasets [9,10], object detection in SAR imagery has become a common and important task drawing broad attention [11]. However, detecting densely packed objects with random orientations (e.g., cars and ships) is challenging. Similar challenges also exist in other application domains. For example, automated rebar counting in construction site images requires detecting densely placed rebars with diverse orientations [12]. Likewise, analyzing building facade characteristics from street view images involves identifying structural elements under varying viewpoints and complex backgrounds [13]. In autonomous driving, datasets such as RSUD20K [14] further highlight these challenges, featuring dense traffic scenes, varying viewpoints, and diverse weather conditions that demand robust object detection.

**Figure 1:**
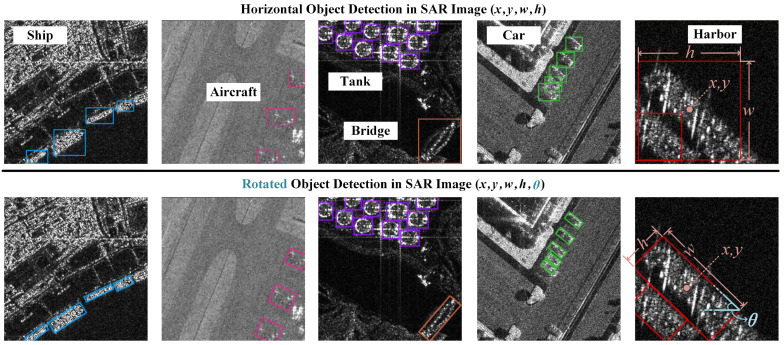
Qualitative comparison between horizontal bounding boxes and rotated bounding boxes in SAR images, illustrating the precision advantage of the latter in localizing arbitrarily oriented ships, slender bridges, dense cars, and overlapping harbors.

These tasks share common challenges with SAR imagery, including orientation variability and background clutter. Traditional detectors use horizontal rectangular boxes [15], which often have too much overlap and poor location accuracy [16]. This problem is worse for slender objects such as bridges, which occupy only a very small part of the detection box. Therefore, rotated object detection in SAR scenes has become an important research problem [17], as illustrated in Figure 1.

Unlike horizontal objects described by [x,y,w,h], as shown in the upper part of Figure 1, rotated objects require an additional prediction of the rotation angle θ. This angle has a decisive influence on detection and localization accuracy [18]. To address this problem, various paradigms for rotated object detection have been developed in the field of computer vision. Early methods often adapted existing region proposal networks such as the R-CNN series [19] for rotation. More recently, efficient single-stage detectors have become more prevalent, e.g., R^3^Det [20], S^2^A-Net [21], etc. These detectors directly regress the parameters of a rotated bounding box. They use either five parameters (center coordinates, width, height, rotation angle) or eight parameters (four corner coordinates). This approach has achieved significant progress in detection performance.

Although these methods have achieved some effectiveness in SAR image object detection [22,23], their original design had a different focus, being primarily targeted at natural optical images. Thus, they typically rely solely on the intensity information of SAR images, neglecting their unique imaging characteristics. In fact, SAR images are generated by active microwave coherent imaging. As such, their frequency domain properties play a crucial role in suppressing speckle noise and enhancing information. Although some research has introduced frequency domain methods into image processing [24], efficient feature enhancement methods are still lacking. Therefore, designing a dedicated detection framework that exploits the properties of SAR imagery is crucial for advancing performance.

As a powerful multi-resolution analysis tool, the wavelet transform offers a frequency domain solution [25]. It can decompose an image into different frequency sub-bands, and the low-frequency features describing the overall structural information of the object are relatively robust to noise. Meanwhile, the high-frequency features capture edge and texture details. These features can help to improve detection precision. Existing research applying the wavelet transform to visual tasks in noisy SAR scenes has achieved good results [26]. Inspired by this, we explore integrating the wavelet transform more deeply into the detection network architecture with the goal of enhancing the model’s feature learning capability for rotated object detection under noise interference.

In addition, the angle parameter is decisive for the final localization accuracy in rotated object detection [27]. Designing more precise angle predictors and loss functions is crucial [28]. Existing methods typically use multi-layer perceptron (MLP) for the prediction heads [29]. MLP performs angle regression through fixed nonlinear combinations of activation functions. However, its expressive capability is constrained. This limitation hinders its effectiveness in modeling key geometric properties such as angle periodicity and boundary discontinuity. This issue becomes particularly pronounced in SAR scenes, where subtle angular deviations can lead to significant IoU degradation.

Fortunately, the recently proposed Kolmogorov–Arnold network [30] offers a new perspective on this challenge. KAN replaces the fixed activation functions in traditional MLP with learnable spline functions. This enables direct function learning on the network connections. Theoretically, it can achieve higher approximation accuracy and interpretability with fewer parameters [31]. This makes it particularly suitable for modeling nonlinear regression problems with strong periodicity and boundary sensitivity, including angle prediction. Inspired by this, we introduce KAN into the rotated object detection framework. We construct a KAN-based angle prediction head with the aim of more finely modeling the complex mapping relationship between object orientation and scattering characteristics. This is expected to significantly improve the stability and accuracy of angle regression.

Based on a thorough analysis of the limitations of existing methods, this paper proposes an SAR image rotated object detection framework named WE-KAN. The main contributions of this work are as follows:

(1) A KAN-based angle prediction head is proposed for rotated object detection in SAR images. By leveraging the learnable spline functions of KAN, this design provides improved modeling of nonlinear angle regression compared to conventional MLP heads. (2) A wavelet domain feature enhancement mechanism is constructed by integrating wavelet scattering coefficients with an attention-based fusion module. Combined with a joint loss function that incorporates rotated IoU and Gaussian distance losses, this design enhances localization precision for slender objects in complex SAR scenes. (3) Excellent performance is achieved on the large-scale RSAR benchmark, with an AP50 of 70.1 and mAP of 35.9, outperforming other existing detection methods.

The rest of this paper is organized as follows: Section 2 reviews the related works, covering wavelet-based feature enhancement and angle representation; Section 3 elaborates on the proposed WE-KAN framework in detail, including its wavelet-based feature extraction module and the KAN-based prediction head; Section 4 presents the experimental setup and quantitative results on public datasets; Section 5 provides in-depth analysis and discussion of the experimental findings, including ablation studies and visualization; finally, Section 6 concludes the paper.

## 2. Related Works

In this section, we review the existing works from three aspects, including rotated object detection frameworks, wavelet-based feature enhancement methods, and angle prediction techniques for rotated objects.

### 2.1. Rotated Object Detection and Loss Functions

Rotated object detection enables higher detection precision. This makes it a key technology for improving the localization of arbitrarily oriented objects such as vehicles and ships [32]. In recent years, research has focused primarily on acquiring better features for rotated objects [20,33]. For instance, S^2^A-Net [21] predicts an initial rotation angle to align convolutional features. It performs more accurate detection based on the aligned features to address the misalignment between rotated objects and features. ReDet [34] proposes a new backbone network called rotation-equivariant networks specifically for rotated objects. It captures their geometric properties in order to achieve high-quality detection.

On the other hand, loss function design is also crucial, since rotated bounding box regression exhibits strong geometric sensitivity. A traditional smooth L1 loss often leads to unstable training and performance saturation. Dedicated loss functions for rotated boxes have been proposed [35,36]. For example, the Gaussian Wasserstein distance loss (GWD loss) [37] takes a novel approach. It models a rotated bounding box as a two-dimensional Gaussian distribution and uses the Wasserstein distance as a metric. This effectively alleviate boundary discontinuity issues caused by angle periodicity. Another mainstream approach is the rotated IoU loss function [38]. It provides geometric supervision by directly computing the differentiable overlap area between predicted and ground truth boxes. These methods have achieved significant progress in both network architectures and loss functions.

However, most existing rotated object detection frameworks have a limitation in that they are designed for natural images such as aerial imagery. Consequently, they do not adequately account for the complex backgrounds and noise present in SAR images. Moreover, advanced loss functions are often treated as independent optimization objectives. In this work, we design a novel rotated object detection framework tailored for SAR images. We also perform deep co-design and joint optimization of a dedicated angle regressor to enhance the angle regression accuracy.

### 2.2. Feature Enhancement Based on Wavelet Transform

The inherent coherent speckle noise and complex backgrounds in SAR images pose a serious challenge to effective feature extraction. The wavelet transform [39] is a classic multi-resolution analysis tool. It has long been used for image feature enhancement and denoising due to its excellent localization properties in the time–frequency domain. Early research established the mathematical foundation of wavelet theory for image decomposition and reconstruction [40]. In the field of remote sensing, Achim et al. designed wavelet-based denoising methods to suppress coherent speckle noise while preserving texture details [41]. These approaches primarily applied wavelet transform in denoising or preprocessing stages.

With the rise of deep learning, researchers have begun to explore integration of the wavelet transform with convolutional neural networks (CNNs) [42]. For example, Wavelet-SRNet [43] is a CNN-embeddable wavelet soft thresholding denoising network that optimizes denoising thresholds through end-to-end learning. This enhances target recognition accuracy in noisy SAR images while at the same time maintaining the model’s robustness to noise. Recently, MSFA [26] analyzed various feature extraction methods on SAR images. It achieved better performance for horizontal object detection methods based on wavelet scatter coefficients [44] through transfer learning.

In fact, deeply integrating wavelet-domain features into an end-to-end object detection framework remains a challenging problem. Specifically, designing effective fusion mechanisms to complement and enhance the wavelet features alongside the original image features merits further research. To this end, we propose a lightweight and efficient multi-branch fusion module with attention mechanisms to tackle this issue. The proposed module embeds multi-scale feature learning from the wavelet domain into the detection network’s backbone with the aim of enhancing the model’s feature discrimination capability in complex SAR scenarios.

### 2.3. Angle Predicting Methods for Rotated Object Detection

Rotated object detection is characterized by the output of an additional angle parameter compared to horizontal bounding boxes. Early research primarily involved improvements based on existing conventional object detection frameworks. For example, RoI Transformer [45] proposed a plug-and-play module that learns a supervised transformation from horizontal boxes to rotated boxes and aligns features. This addresses the core issue of misalignment between rotated objects and features in aerial images, significantly improving the accuracy of rotated object detection. In practice, a more direct approach is to design a separate branch dedicated to angle prediction [46]. Such methods have been applied in single-stage detectors such as FCOS [47] and RetinaNet [48] as well as in two-stage frameworks such as Faster R-CNN [15]. Moreover, specialized angle encoders have been designed to address angle periodicity and boundary discontinuity. Examples include ACM [18] and PSC [27], which are based on 2D and 3D spaces, respectively. In fact, the search for a reliable angle predictor constitutes a long-standing and active area of research.

Compared with traditional MLP-based regression methods, transformer-based detectors [49] offer new insights for angle prediction. AO2-DETR [50] was one of the earliest works to introduce DETR into rotated object detection. It uses adaptive oriented proposals with global attention. RS-DETR [51] further introduced a rotation–semantic co-attention module to jointly learn orientation and semantic features. ARS-DETR [33] focused on aspect-ratio sensitivity with a long-edge definition strategy. Recently, a different approach has involved Fourier angle alignment [52] by transforming features into the frequency domain to align their orientations. Despite impressive performance, these transformer methods rely on self-attention mechanisms that significantly increase computational cost, leaving room for the development of more efficient architectures.

In contrast to existing approaches, the recently proposed KAN [30] offers a new network architecture paradigm for complex function regression. Unlike both MLPs and transformers, which rely on fixed activation functions at nodes, KAN employs learnable spline functions on the edges of the network. Theoretically, this can achieve higher approximation accuracy and model interpretability with fewer parameters. This characteristic makes KAN particularly suitable for modeling problems with strong nonlinearity and periodic patterns. Currently, KAN has undergone preliminary exploration in vision tasks, showing considerable progress in both recognition [53] and segmentation tasks [54]. More recently, U-KABS [55] integrated KAN with a U-shaped architecture for medical image segmentation, combining Bernstein polynomials for global smoothness and B-splines for local adaptability. This further underscores KAN’s potential for dense prediction tasks requiring precise boundary delineation. Moreover, Ding et al. [56] proposed DSKFuse, a multimodal image fusion framework. They integrated KAN with dynamic sparse transformers and achieved state-of-the-art performance in both fusion quality and downstream tasks, including object detection and segmentation. These works validate KAN’s capability in modeling nonlinear geometric relationships, providing a potential foundation for its application in the current domain.

Inspired by this, the flexible function learning capability of KAN offers a new approach to tackling the challenge of angle regression for rotated bounding boxes. It holds promise for constructing a more powerful and robust angle prediction module in the rotated object detection task. Therefore, in this work we design a KAN-based angle prediction head. Its function is to more finely model the complex mapping relationship between object orientation and image features.

## 3. Materials and Methods

**Figure 2:**
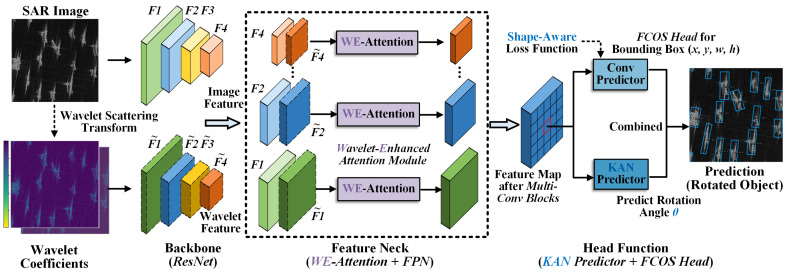
Overall architecture of the WE-KAN rotated object detection method.

This section provides a detailed description of the proposed method for rotated object detection in SAR images. WE-KAN is a novel framework that employs wavelet transformation for feature enhancement and incorporates KAN for angle prediction. The method consists of a backbone network, a feature neck, and a head function. The overall flowchart is shown in Figure 2.

The overall architecture of WE-KAN is depicted in Figure 2. The framework takes two parallel inputs: the original SAR intensity image, and its corresponding wavelet scatter coefficients. These are processed independently through backbone to extract multi-scale feature representations, denoted as $\left\{F_{1},F_{2},\ldots ,F_{4}\right\}$ and $\left\{{\tilde{F}}_{1},{\tilde{F}}_{2},\ldots ,{\tilde{F}}_{4}\right\}$, respectively. At each scale within the feature neck, we introduce a lightweight fusion module called WE-Attention. This module dynamically integrates the SAR-derived features F and wavelet-enhanced features $\tilde{F}$ via attention mechanisms.

The fused multi-scale features are then forwarded to a detection head based on FCOS, which regresses the center coordinates, width, and height of bounding box, i.e., [x,y,w,h]. In parallel, a dedicated branch built upon the Kolmogorov–Arnold network is employed to predict the rotation angle θ. The combination of these outputs forms the complete rotated bounding box representation as [x,y,w,h,θ]. The remainder of this section elaborates on the key components of WE-KAN.

### 3.1. Wavelet Coefficients and the WE-Attention Module

The inherent coherent speckle noise in SAR images interferes with intensity-based feature extraction, posing challenges for subsequent detection tasks. Constructing auxiliary features is a solution that can enhance detection accuracy. The wavelet transform serves as a classical time-frequency analysis tool capable of effectively decoupling image signals into different frequency bands. Therefore, our method employs the wavelet transform to extract auxiliary features. Its formulation can be expressed as follows:

(1) $${\mathrm{Wave}}_{f}\left(a,b\right)=\frac{1}{\sqrt{a}}\int_{-\infty }^{+\infty }f\left(t\right)\cdot \psi \left(\frac{t-b}{a}\right)dt$$

where *a* is the scale parameter controlling the frequency range of analysis, *b* is the translation parameter, and ψ(t) is the mother wavelet function.

However, the continuous form of the wavelet transform cannot be directly applied to image signals. For auxiliary features used in learning, a more common practice is to extract wavelet scatter coefficients [44], which exhibit better robustness to noise. Specifically, to enhance directional sensitivity, an eight-direction scattering transform is employed here. It applies a modulus nonlinearity to the wavelet coefficients followed by low-pass filtering, yielding a feature representation. This representation effectively captures multi-directional texture patterns, providing a discriminative feature for the detection stages. For a two-dimensional image I(x,y), its wavelet scatter coefficients are as follows:

(2) $$\tilde{F}=\left[\begin{array}{c} S_{0}I\left(x,y\right)=\left(I*h\right)\left(x,y\right)\\ S_{1}I\left(x,y,\theta_{1}\right)=\left(\left|I*g_{\theta_{1}}\right|*h\right)\left(x,y\right)\\ \ldots \\ S_{1}I\left(x,y,\theta_{8}\right)=\left(\left|I*g_{\theta_{8}}\right|*h\right)\left(x,y\right) \end{array}\right]\in R^{H\times W\times 9},$$

where h denotes the low-pass filter, $g_{\theta }$ is the wavelet filter in the θ direction, |·| represents the modulus operation, and ∗ denotes the convolution operation.

**Figure 3:**
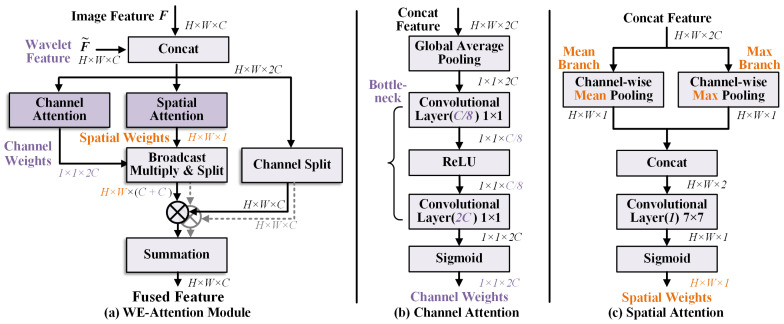
The proposed WE-Attention module for fusing SAR and corresponding wavelet features: (**a**) overall structure of WE-Attention, (**b**) structure of the channel attention module, and (**c**) structure of the spatial attention module.

To fully utilize the original SAR intensity information while preserving the wavelet scattering features, this work takes a specific approach. We employ dual independent backbone networks for parallel feature extraction, as illustrated in Figure 2. These two backbone networks share the same structure to ensure feature scale consistency. The extracted multi-scale features are denoted as $\left\{F_{1},F_{2},\ldots ,F_{4}\right\}$ and $\left\{{\tilde{F}}_{1},{\tilde{F}}_{2},\ldots ,{\tilde{F}}_{4}\right\}$, respectively. Features from both branches remain spatially aligned at the same scale, facilitating cross-modal feature fusion and interaction. For each feature level, we design an attention-based fusion module, as shown in Figure 3.

In Figure 3, we design two types of attention mechanisms to achieve fusion. Specifically, the image feature F and the wavelet feature $\tilde{F}$ are concatenated along the channel dimension to form a tensor of size H×W×2C. This tensor is then fed separately into a channel attention layer and a spatial attention layer. The former generates channel weights of size 1×1×2C through global pooling and a bottleneck structure, while the latter employs a channel-wise pooling strategy to produce spatial weights of size H×W×1. The channel and spatial weights are combined via element-wise multiplication to produce the final fused feature, as expressed below:

(3) $$\mathrm{WEA}\left(F,\tilde{F}\right)=F\cdot {\left(\mathrm{CA}⊙\mathrm{SA}\right)}_{F}+\tilde{F}\cdot {\left(\mathrm{CA}⊙\mathrm{SA}\right)}_{\tilde{F}}$$

where WEA(·,·) means the proposed wavelet-enhanced attention, ⊙ denotes the element-wise (Hadamard) multiplication, and CA, SA respectively represent the channel and spatial attention.

The subscripts F and $\tilde{F}$ in (3) indicate the respective weights for the original SAR features and the wavelet features. This design enables the network to dynamically determine the fusion strategy for the two types of features, allowing the network to adaptively emphasize the more informative modality at each spatial location and channel. For example, in regions dominated by speckle noise, the network could behave differently by assigning higher weights to wavelet features due to their denoising capability. Conversely, in texture-rich areas, original SAR features might contribute more. This adaptive weighting mechanism is able to realize the complementary strengths of both modalities.

Furthermore, incorporating bottleneck and pooling operations significantly reduces computational overhead. The channel attention module first compresses the concatenated features through global average pooling, then applies two fully connected layers with a reduction ratio to limit the parameter count. Similarly, the spatial attention module employs channel-wise pooling to aggregate information before generating spatial weights. These design choices result in a more lightweight feature fusion structure, enhancing representation learning without introducing excessive computational burden. This makes our WE-Attention module suitable for practical SAR image analysis tasks.

### 3.2. KAN-Based Angle Predictor

In rotated object detection, predicting the angle parameter θ is crucial for determining detection accuracy. Current detectors commonly use multi-layer perceptrons (MLPs) or conventional convolutional networks as prediction heads [29]. However, angles exhibit strong periodicity challenges, such as the boundary discontinuity between −π/2 and +π/2. For targets with high aspect ratios, even slight angle variations can lead to significant accuracy changes. MLPs often require stacking more layers and more parameters to address such strong nonlinear mapping problems.

To address these issues, we adopt KAN [30] as a dedicated predictor for angle regression in SAR rotated object detection. This novel network based on the Kolmogorov–Arnold theorem uses learnable smooth spline functions as its activation functions to enhance the network’s nonlinear mapping capability [30]. Typically, it is composed of several Kolmogorov–Arnold layers (KAL). For a 2D input feature F which is flattened (or reshaped) from the spatial tensor into dimensions HW×C, the output at node *j* is expressed as follows:

(4) $$\mathrm{KAL}{\left(F\right)}_{j}=\sum_{i}\phi_{ij}\left(F_{i}\right)$$

where $\phi_{ij}$ is a learnable unary function connecting the $i^{th}$ input to the $j^{th}$ output, usually implemented in the following form:

(5) $$\phi_{ij}\left(F_{i}\right)=p_{b}\cdot \mathrm{SiLU}\left(F_{i}\right)+p_{s}\cdot \mathrm{Spline}\left(F_{i};K_{ij}\right),$$

where $p_{b}$ and $p_{s}$ are learnable scalars that balance the contribution of the basis function and the spline function, $K_{ij}$ is the learnable spline coefficient vector associated with the connection from the $i^{th}$ input to the $j^{th}$ output, Spline(·) is a B-spline function, and SiLU(·) is the Sigmoid linear unit activation function.

In (5), the first term serves as the basis function, ensuring global approximation capability by maintaining a residual connection to the input. This design choice prevents the network from relying entirely on the learnable splines and helps to stabilize early-stage training. The second term is a learnable B-spline function used for flexibly fitting local nonlinear variations. This dual-component design allows KAN to capture both coarse and fine-grained patterns in the angle regression task. The spline function is formed by combining a set of learnable coefficients $K_{ij}=\left[K_{ij,1},\ldots ,K_{ij,G}\right]$ with B-spline basis functions $B_{g}$:

(6) $$\mathrm{Spline}\left(F_{i};K_{ij}\right)=\sum_{g=1}^{G}K_{ij,g}\cdot B_{g}\left(F_{i}\right)$$

where *G* is determined by the spline order and grid size.

The superiority of KAN for angle regression stems from its mathematical properties. Angle prediction in rotated object detection presents unique challenges. First, angles exhibit inherent periodicity with boundary discontinuity at ±π/2, which is a complex nonlinear mapping between feature representations and angular values. Second, for slender objects such as ships and bridges, small angular deviations can lead to significant IoU degradation. Therefore, angle prediction requires careful design.

KAN addresses these challenges through its learnable spline-based architecture. Unlike MLP, which uses fixed activation functions at nodes, KAN parameterizes the edges with learnable B-spline functions. As formulated in (5), the combination of a residual basis function and learnable splines enables both global approximation capability and flexible local adaptation. This is particularly advantageous for angle regression. The mapping function may be smooth in some regions but still require rapid adjustments near boundary discontinuities. The learnable spline functions can adapt to the local variations of the angle regression task. This allows KAN to achieve higher precision than conventional MLP-based approaches.

**Figure 4:**
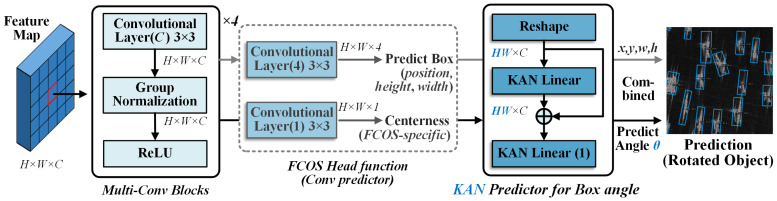
Proposed rotation detection head based on KAN predictor for box angle.

To implement rotated object detection, we design a lightweight and efficient prediction head that follows the extracted fused feature maps. It contains several convolutional blocks and adds a dedicated KAN predictor alongside the existing FCOS head. The FCOS head is responsible for predicting conventional bounding box parameters including center coordinates, width, height, and centerness score. Meanwhile, the KAN predictor is specifically designed for bounding box angle prediction. This decoupled architecture allows each branch to focus on its respective regression task, as illustrated in Figure 4.

In Figure 4, the fused feature map is a three-dimensional tensor $F\in R^{H\times W\times C}$. To meet the input requirements of the KAN predictor, we flatten and reshape it into a two-dimensional feature matrix $F_{\mathrm{flat}}\in R^{(HW)\times C}$. In this matrix, each row corresponds to the feature vector of a spatial location. To enhance training stability, we incorporate residual connections in the KAN structure as follows:

(7) $$\mathrm{KAN}\left(F_{\mathrm{flat}}\right)=\mathrm{KAL}\left(F_{\mathrm{flat}}+\mathrm{KAL}\left(F_{\mathrm{flat}}\right)\right).$$

Specifically, the flattened feature $F_{\mathrm{flat}}$ is first fed into a KAL layer. The output of this layer is then added to the original input through a residual connection. This combined signal passes through another KAL layer with an output dimension of 1, which finally produces the predicted rotation angle θ. Together with the center coordinates, width, and height predicted by the FCOS head, this angle θ forms the complete set of rotated object parameters for detection.

### 3.3. Loss Function Design of WE-KAN

During the training phase, WE-KAN inherits the FCOS object detection framework. Its loss function consists of three components: classification loss, centerness loss, and regression loss. It is defined as follows:

(8) $$\begin{array}{c} L_{\mathrm{Total}}=\frac{1}{\mathrm{Pos}}\sum_{i}^{\mathrm{Pos}}\left[L_{\mathrm{Cls}}\left(p_{i},gt_{i}^{*}\right)+L_{\mathrm{Centerness}}\left(d_{i},d_{i}^{*}\right)+L_{\mathrm{Box}}\left(t_{i},t_{i}^{*}\right)\right], \end{array}$$

where Pos is the number of positive samples, $p_{i}$ is the predicted score for the detected object, $d_{i}$ is the predicted centerness, $d_{i}^{*}$ is the actual centerness, $gt_{i}^{*}$ is the true category label, $t_{i}=\left[x,y,w,h,\theta \right]$ are the predicted rotated bounding box parameters, and $t_{i}^{*}$ are the true bounding box parameters.

In (8), the first term is responsible for predicting the object category and the second term is the centerness loss for predicting confidence. In the implementation, the classification loss and centerness loss adopt the focal loss [48] and cross-entropy loss, respectively, as shown below:

(9) $$L_{\mathrm{Cls}}\left(p_{i},gt_{i}^{*}\right)=\left\{\begin{array}{cc} -\alpha {\left(1-p_{i}\right)}^{\gamma }\log\left(p_{i}\right) & gt_{i}^{*}=1\\ -\left(1-\alpha \right)p_{i}^{\gamma }\log\left(1-p_{i}\right) & gt_{i}^{*}=0 \end{array}\right.,$$

(10) $$L_{\mathrm{Centerness}}\left(d_{i},d_{i}^{*}\right)=-\left[d_{i}^{*}\log\left(d_{i}\right)+\left(1-d_{i}^{*}\right)\log\left(1-d_{i}\right)\right],$$

where α=0.25 denotes the class balancing parameter, γ=2 is the focusing parameter, and the centerness $d_{i},d_{i}^{*}$ can be directly calculated from the distances of the four sides of the bounding box.

For the bounding box loss $L_{\mathrm{Box}}\left(t_{i},t_{i}^{*}\right)$ in (8), we adopt a shape-aware fused loss. The challenge in rotated object detection lies in the strong coupling of bounding box parameters. Independent parameter regression (such as the smooth L1 loss) may lead to inconsistency between optimization objectives and evaluation metrics (e.g., IoU). To address this, we design a regression loss function that combines geometric overlap and distribution similarity.

First, the most direct geometric supervision is to maximize the intersection over union between the predicted rotated bounding box ${\mathrm{RB}}_{t}$ and the ground truth rotated bounding box ${\mathrm{RB}}_{t^{*}}$. This is also common in conventional horizontal object detection tasks. The loss is defined as follows:

(11) $$L_{\mathrm{RIoU}}\left(t,t^{*}\right)=1-\frac{\mathrm{Area}({\mathrm{RB}}_{t}\cap {\mathrm{RB}}_{t^{*}})}{\mathrm{Area}({\mathrm{RB}}_{t}\cup {\mathrm{RB}}_{t^{*}})}.$$

However, the rotated IoU loss provides weaker gradient signals when bounding boxes do not overlap or only slightly overlap. This is especially true in dense or small-object scenarios. To compensate for this limitation, we introduce a supervision metric based on probability distribution [37]. It parameterizes a rotated rectangular box as a two-dimensional Gaussian distribution $N\left(\mu_{t},\Sigma_{t}\right)$, where the mean $\mu_{t}$ corresponds to the box center coordinates and the covariance matrix $\Sigma_{t}$ is determined by the box width *w*, height *h*, and rotation angle θ as shown below:

(12) $$\mu =\left[\begin{array}{c} x\\ y \end{array}\right],\;\Sigma =R_{\theta }\left[\begin{array}{cc} \frac{w^{2}}{4} & 0\\ 0 & \frac{h^{2}}{4} \end{array}\right]R_{\theta }^{T}$$

where $R_{\theta }=\left[\begin{array}{cc} \cos\theta & -\sin\theta \\ \sin\theta & \cos\theta \end{array}\right]$ is the rotation matrix determined by the angle θ.

At this point, the ground truth and predicted bounding boxes become two Gaussian distributions. We can then construct the loss function by measuring the distance between these distributions:

(13) $$L_{\mathrm{GD}}\left(t,t^{*}\right)=\log\left(1+\sqrt{d^{2}\left(N\left(\mu_{t},\Sigma_{t}\right),N\left(\mu_{t}^{*},\Sigma_{t}^{*}\right)\right)}\right),$$

where $d^{2}\left(\cdot ,\cdot \right)$ represents the squared second-order Wasserstein distance between the two distributions, expressed as follows:

(14) $$\begin{array}{c} d^{2}\left(N\left(\mu_{t},\Sigma_{t}\right),N\left(\mu_{t}^{*},\Sigma_{t}^{*}\right)\right)={∥\mu_{t}-\mu_{t}^{*}∥}^{2}+{∥\Sigma_{t}^{1/2}-\Sigma_{t}^{*1/2}∥}_{F}^{2}, \end{array}$$

where ${∥\cdot ∥}_{F}$ denotes the Frobenius norm.

In (14), the first term based on the means represents the distance between centers, while the second term based on the covariance reflects the shape difference between the distributions. Compared to $L_{\mathrm{RIoU}}$, which focuses on maximizing macroscopic geometric overlap, $L_{\mathrm{GD}}$ emphasizes conveying the shape information of the object. To leverage the advantages of both losses, we design a fused shape-aware loss function, as follows:

(15) $$L_{\mathrm{Box}}\left(t_{i},t_{i}^{*}\right)=\tau \cdot L_{\mathrm{RIoU}}\left(t,t^{*}\right)+\left(1-\tau \right)\cdot L_{\mathrm{GD}}\left(t,t^{*}\right)$$

where τ is a hyperparameter used to balance the two terms.

In (15), $L_{\mathrm{RIoU}}$ ensures overall localization of the detected box while $L_{\mathrm{GD}}$ tunes its orientation and shape. The two complement each other during optimization. This enables the model to achieve precise and stable rotated box regression performance in challenging SAR images.

## 4. Results

In this section, we conduct a comprehensive evaluation of the proposed WE-KAN method for SAR images. Section 4.1 introduces the dataset and evaluation metrics used in our experiments, Section 4.2 details the implementation settings, including network configuration and training parameters, and Section 4.3 presents the main experimental results before comparing our method with state-of-the-art approaches on the RSAR dataset.

### 4.1. Dataset and Evaluation Metrics

To validate the effectiveness of the proposed method, this study adopts the recently released RSAR dataset [17] for training and evaluation. This dataset is derived from the integration of ten typical SAR datasets, building upon the foundation of Sardet-100k [26]. It is currently one of the largest publicly available multi-category rotated SAR object detection datasets. The dataset comprises 95,842 images with 183,534 instances. These instances span six categories: aircraft, bridge, car, harbor, ship, and tank. According to the established benchmark, the dataset is partitioned into 78,837 training samples, 8467 validation samples, and 8538 testing samples.

The dataset presents two major challenges for rotated object detection. First, it exhibits significant category imbalance, with ship instances accounting for 62.19% of all annotations and harbor instances representing only 2.24%. Second, object scales vary dramatically across categories. Harbors typically occupy large spatial regions, whereas tanks and cars are often represented by only a few pixels. This combination of factors creates a demanding environment. The dataset contains both a long-tail distribution and extreme scale variation. It demands detectors that are both robust to minority classes and sensitive to objects of vastly different sizes.

For quantitative analysis, we adopt the average precision (AP) as the evaluation metric. AP is a widely used metric in object detection [57]. It is computed as the area under the precision–recall curve, with higher values indicating better detection performance across confidence thresholds. In rotated object detection, IoU calculation incorporates angle information, providing a comprehensive measure of both box localization and angle regression accuracy. To ensure fair comparison, all reported results are evaluated on the official test set.

### 4.2. Implementation Details

The proposed method employs the *le90* (long-edge-90) format to represent rotated objects, where the rotation angle θ is defined within the range from −π/2 to +π/2. For wavelet scattering coefficient extraction, we use the Kymatio library [58] with J=1 (decomposition levels). Kymatio provides GPU-accelerated wavelet scattering transforms, which enables seamless integration into deep learning pipelines. The scattering transform generates nine-dimensional coefficients per pixel. These include one low-frequency component and L=8 directional components. The extracted wavelet features are processed by a separate backbone with the number of input channels set to 9. They are then fused with the original image features through attention modules at multiple scales. Under the baseline configuration, the model is trained on the training set for 12 epochs before being evaluated on the test set.

Regarding model architecture, our framework consists of a backbone network, a feature neck, and a prediction head. Using ResNet50 [59] as the backbone, features are extracted at four scales with channel dimensions of 256, 512, 1024, and 2048, respectively. These multi-scale features are then processed through the proposed WE-Attention module and FPN [60] architecture, which unify the channel dimension to 256 at each scale. This unified dimension maintains a balance between representational capacity and computational efficiency. For the KAN-based prediction head, we set the grid size to 5 and the spline order to 3 in order to achieve optimal angle regression performance. The loss function hyperparameter τ in Equation (15) is set to 0.5 in order to balance the contributions of the rotated IoU loss $L_{\mathrm{RIoU}}$ and Gaussian distance loss $L_{\mathrm{GD}}$.

To ensure fair comparison with existing methods, we adopt the same experimental settings as the MMRotate benchmark [46]. All models are trained for 12 epochs using stochastic gradient descent (SGD) with an initial learning rate of 0.0025, momentum of 0.9, and weight decay of 0.0001. The learning rate follows a linear warm-up schedule for the first 500 iterations, followed by multi-step decay at epochs 8 and 11 with a gamma of 0.1. During inference, we apply rotated NMS with an IoU threshold of 0.1 and keep up to 2000 detections per image. The input images are resized and padded to 800 × 800 pixels while preserving the aspect ratio. All experiments are conducted on an NVIDIA RTX 5880 Ada Generation (NVIDIA Corporation, Santa Clara, CA, USA) graphics card using the MMRotate framework.

### 4.3. Performance of Rotated Object Detection

This section validates the proposed method against existing approaches. The compared methods include single-stage approaches such as Rotated-RetinaNet [48] and S^2^A-Net [21], two-stage methods such as O-RCNN [19] and ReDet [34], and transformer-based methods [33]. Following this setup, we present the quantitative results in Table 1. It summarizes the overall performance metrics (AP50, AP75, and mAP) across all methods, with the top two results in each column highlighted. A detailed category-wise performance analysis is provided in the discussion section.

Table 1 presents the overall performance comparison between WE-KAN and existing methods on the RSAR dataset. With the ResNet50 backbone and 1× training schedule, our method achieves the highest AP50 score of 70.1 and a mAP of 35.9, outperforming all compared approaches. Specifically, we compare it to the baseline Rotated-FCOS method, which scores 66.7 AP50 and 34.2 mAP. WE-KAN shows clear improvement, improving AP50 by 3.4 points and mAP by 1.7 points. This demonstrates the effectiveness of our proposed wavelet feature enhancement and KAN predictor. Against the strong single-stage detector S^2^A-Net (66.5 AP50, 33.1 mAP), our method achieves gains of 3.6 points in AP50 and 2.8 points in mAP. When compared to the two-stage O-RCNN (64.8 AP50, 33.6 mAP), WE-KAN shows improvements of 5.3 points in AP50 and 2.3 points in mAP. Even against RoI-Transformer (66.9 AP50, 35.0 mAP), which achieves the second-best mAP among existing methods, WE-KAN still maintains advantages of 3.2 points in AP50 and 0.9 points in mAP. Notably, ReDet outperforms our method in terms of the AP75 metric (32.8 vs. 32.5). However, on the primary evaluation metrics of AP50 and mAP, WE-KAN demonstrates superior performance, indicating better overall detection accuracy.

We also explore scaling up the model. When using a ResNet101 backbone and extending the training schedule to 3×, the performance of WE-KAN improves further and significantly, achieving an AP50 of 74.6 and a mAP of 37.5. This represents substantial gains of 4.5 points in AP50 and 1.6 points in mAP over the ResNet50 configuration. The consistent performance improvement across different architectures and training scales fully demonstrates the scalability and stability of our proposed method.

Synthetic aperture radar image analysis faces several inherent challenges, including strong speckle noise interference, diverse object orientations, and complex background scenes. The superior performance of WE-KAN validates its stronger adaptability and robustness in addressing these challenges. This effectiveness stems from the synergistic integration of two key innovations. The wavelet domain feature enhancement provides effective noise suppression and structural perception. Meanwhile, the KAN-based predictor enables accurate angle regression. Together, these innovations enable WE-KAN to achieve balanced and superior performance in SAR image rotated object detection tasks.

## 5. Discussion

In this section, we provide an in-depth analysis and discussion of the proposed WE-KAN method for SAR images. We begin with a category-wise performance analysis and evaluation on the RSDD-SAR dataset in Section 5.1. Section 5.2 investigates the sensitivity of key hyperparameters. The training dynamics of our model are explored in Section 5.3. Section 5.4 and Section 5.5 present ablation studies and inference speed. Section 5.6 visualization analysis to validate each component. Finally, Section 5.7 discusses limitations and future work.

### 5.1. Category-Wise Performance Analysis and Evaluation on RSDD-SAR Dataset

To gain deeper insights into the detection capabilities of different methods, we analyze their performance across the six object categories in the RSAR dataset. Table 2 presents the AP50 scores for the aircraft, bridge, car, harbor, ship, and tank categories. These categories exhibit significant diversity. They differ in object size, aspect ratio, scattering characteristics, and background complexity. This diversity provides a comprehensive benchmark for evaluating rotated object detectors in SAR imagery.

Table 2 reveals significant performance variations across different object categories. For slender targets such as bridges, our WE-KAN method achieves the best results, with 62.1 AP50 when using ResNet50 and 64.8 with ResNet101. This outperforms the next-best method S^2^ANet (60.2) by 1.9 and 4.6 points, respectively, and exceeds the baseline Rotated-FCOS (58.8) by 3.3 and 6.0 points. The improvement stems from two key components. First, wavelet scatter features enhance structural perception by preserving edge information. Second, the KAN predictor provides more accurate angle regression for elongated objects, where small angular errors can cause large IoU drops.

Our method also shows strong results on challenging object categories. For harbor scenes, which typically occupy large spatial regions with complex structures, WE-KAN achieves 74.3 with ResNet50, substantially outperforming all other methods. For tank targets, our method attains 40.8 with ResNet50, representing an improvement of 6.9 points over the baseline. These results demonstrate the effectiveness of our approach. Wavelet domain features effectively suppress background noise while preserving discriminative information. Simultaneously, the KAN predictor ensures precise orientation estimation. The combination of these components contributes to the overall AP50 improvement of 3.4 points over Rotated-FCOS, reaching 70.1 AP50.

To further evaluate the effectiveness of our method, we conduct additional experiments on the RSDD-SAR dataset [16]. This is a publicly available rotated dataset for ship detection in SAR images. It consists of 7000 image slices and 10,263 ship instances, which are derived from 84 Gaofen-3 and 41 TerraSAR-X scenes. The dataset covers diverse scenarios, including inshore and offshore scenes, while the objects exhibit arbitrary orientations, large aspect ratios, and a high proportion of small objects. In the experiments, the hyperparameters remain identical to our RSAR setup and the results are reported in Table 3.

Table 3 reports the comparison results on RSDD-SAR under the ResNet50 backbone. To be specific, our WE-KAN achieves 66.7 inshore AP50, the highest among other methods. This improvement is attributed to our wavelet domain feature enhancement. It effectively suppresses complex background clutter from harbor facilities and land structures, which is a key challenge in inshore ship detection. For offshore scenes, WE-KAN achieves 90.3 AP50, slightly lower than the best-performing methods (RoI-Transformer with 94.4 and Rotated-FCOS with 93.1). This tradeoff may be explained by the attention mechanism in our fusion module. It learns to prioritize challenging inshore samples during training, which helps to maintain overall detection accuracy across both inshore and offshore scenes.

Despite this tradeoff, WE-KAN achieves an overall AP50 of 89.4. This matches the best result (CFA) and outperforms other detectors under the same ResNet50 backbone, including S^2^ANet (87.9 AP50) and Oriented R-CNN (88.8 AP50). The overall performance combined with the significant gain on the more challenging inshore scenes validates the robustness and generalization of WE-KAN. This demonstrates its effectiveness across different SAR datasets and target distributions.

### 5.2. Parameter Sensitivity Analysis

This section presents a sensitivity analysis of the hyperparameter τ. More specifically, the training of our WE-KAN method employs a joint loss function design that integrates the overlap-based $L_{\mathrm{RIoU}}$ and the Gaussian distance loss $L_{\mathrm{GD}}$ through a balancing coefficient τ. To investigate the impact of the balancing coefficient τ on model performance, we conduct a systematic parameter sensitivity study. Specifically, we vary τ from 0.1 to 0.9 in increments of 0.1 while keeping all other experimental settings fixed. The corresponding AP scores under different τ value are reported in Table 4.

In Table 4, a clear correlation can be observed between the weight parameter τ and model performance. When τ=0.1, the model relies excessively on the Gaussian distance loss $L_{\mathrm{GD}}$. Although it performs well in shape alignment, the optimization of geometric overlap is insufficient, resulting in AP50 (69.5) and mAP (35.3) not reaching their optimal values. As τ gradually increases, the weight of the rotated IoU loss $L_{\mathrm{RIoU}}$ rises and the model’s localization accuracy progressively improves, peaking at τ=0.5. This indicates that when the two components are balanced, geometric and distributional supervision can complement each other effectively.

However, when τ continues to increase to 0.9, the model overly emphasizes $L_{\mathrm{RIoU}}$, weakening its ability to finely model angles and shapes, leading to performance degradation. The overall trend shows that the model performs most robustly when τ lies within the interval [0.4, 0.6]. This highlights the critical influence on final detection accuracy of balancing the two components in the joint loss.

We further investigate the impact of wavelet configuration on detection performance. Two key parameters are evaluated: the number of decomposition levels (*J*), and the number of directional filters (*L*). *J* controls the scale of wavelet decomposition, with larger values capturing more global information, while *L* determines the orientation resolution, with higher values providing finer directional sensitivity. The results are reported in Table 5.

The results reveal several insights. First, orientation resolution plays an important role. Configurations with L=8 consistently outperform those with L=4 across both decomposition levels. This indicates that finer directional sensitivity helps to capture object orientation. However, increasing *L* to 16 leads to a slight performance drop. This suggests potential overfitting to directional patterns that may not generalize well.

Second, decomposition level shows a similar trend. J=2 achieves marginally better results than J=1 when combined with L=4 or L=8, but the improvement is modest. More complex configurations with J=2 and L=16 do not yield further gains. In fact, performance slightly degrades compared to simpler settings. Considering the additional computational cost, the improvements from more complex wavelet settings are not substantial. The channel dimension increases significantly (from 9 to 81 or even 289), leading to higher model capacity and longer inference times. Therefore, the configuration with J=1 or J=2 and L=8 offers a more practical balance between performance and efficiency.

### 5.3. Training Dynamics Analysis

**Figure 5:**
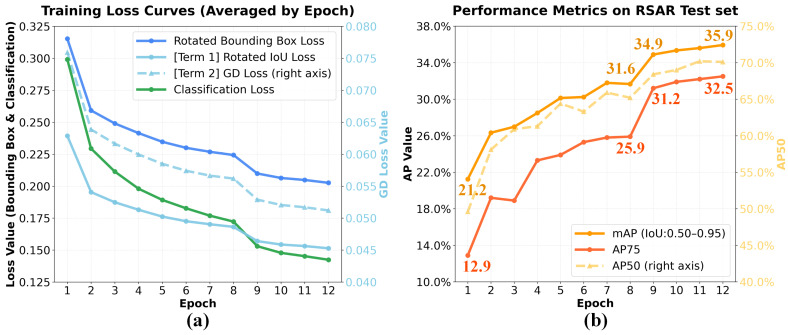
Training dynamics under the optimal configuration (τ=0.5): (**a**) evolution of the training losses, including classification loss and box loss with its two terms (rotated IoU loss, GD loss), and (**b**) corresponding evaluation metrics on the test set, including AP50, AP75, and mAP.

The proposed joint loss function integrates geometric overlap ($L_{\mathrm{RIoU}}$) and distribution similarity ($L_{\mathrm{GD}}$) to guide rotated bounding box regression. To better analyze these two components evolve during training, Figure 5 presents the loss curves and AP scores during model training when τ=0.5.

In Figure 5, it can be seen that the downward trends of $L_{\mathrm{RIoU}}$ and $L_{\mathrm{GD}}$ are generally consistent. This indicates that the two loss components are optimized cooperatively during training, without significant gradient conflict or dominance. Meanwhile, the overall bounding box loss $L_{\mathrm{Box}}$ and classification loss $L_{\mathrm{Cls}}$ also exhibit stable declines. This demonstrating demonstrates the model’s good convergence stability under the multi-task learning framework. Regarding AP scores, the model progressively improves from an initial mAP of 21.2, reaching 34.9 by epoch 9 of training and ultimately achieving 35.9 mAP by the end of epoch 12. The notable improvement at epoch 9 is primarily due to the learning rate decay strategy in the training schedule. This trend aligns with the variations in the loss functions. Overall, the loss curves are smooth and the AP growth is stable. This indicates that WE-KAN exhibits favorable dynamic characteristics during training, laying a solid foundation for enhancing model prediction accuracy.

### 5.4. Ablation Study

Our proposed WE-KAN incorporates three main components: wavelet feature enhancement, a KAN predictor, and a fused loss function. To validate the effectiveness of each component in WE-KAN, this section presents ablation studies focusing on the input features, predictor, and loss function design. All experiments are performed using ResNet50 as the backbone network under identical training settings. Accordingly, the quantitative results are presented in Table 6.

In Table 6, the baseline method uses only the original SAR image as input and employs an MLP for angle prediction with the $L_{\mathrm{RIoU}}$ loss, achieving 66.7 AP50 and 34.2 mAP. When replacing the loss function with $L_{\mathrm{GD}}$ alone, we observe an increase in AP50 to 67.6 but a drop in AP75 from 31.5 to 29.4, resulting in a slightly lower mAP of 33.9. This suggests that while the Gaussian distance loss helps with coarse localization, it is less effective for fine-grained alignment compared to the rotated IoU loss.

To leverage the complementary strengths of both loss functions, we first evaluate the fusion loss combining $L_{\mathrm{RIoU}}$ and $L_{\mathrm{GD}}$. This configuration improves all metrics, achieving 68.7 AP50, 32.0 AP75, and 34.7 mAP. The consistent gains across all three indicators confirm that the two loss components optimize different aspects of bounding box regression and work synergistically when combined.

We then examine the contribution of wavelet feature enhancement. Introducing wavelet scattering coefficients as additional input while keeping the MLP predictor and $L_{\mathrm{RIoU}}$ loss yields 69.0 AP50 and 35.0 mAP. This demonstrates that wavelet domain features effectively suppress speckle noise and enhance structural perception.

The ablation results in Table 6 provide experimental evidence for KAN’s practical advantages. Replacing the MLP predictor with KAN while keeping all other components fixed improves AP50 from 66.7 to 68.1. AP75 increases from 31.5 to 32.7. Notably, the gain in AP75 (1.2 points) is comparable to the gain in AP50 (1.4 points). This indicates that KAN’s benefit is particularly pronounced at stricter IoU thresholds, where precise angle alignment is essential. This result aligns with the theoretical expectation. KAN’s learnable spline functions can better model the nonlinear mapping between SAR features and orientation angles, especially near boundary discontinuities.

Building upon these findings, we combine wavelet features with the KAN predictor under different loss configurations. Using $L_{\mathrm{RIoU}}$ alone achieves 68.9 AP50 and 35.5 mAP, while $L_{\mathrm{GD}}$ alone reaches 69.3 AP50 but a lower 32.0 AP75 and 35.0 mAP. The tradeoff observed here further highlights the importance of balanced supervision.

Finally, our full WE-KAN configuration integrates all three proposed components: wavelet feature enhancement, the KAN predictor, and the joint loss function combining $L_{\mathrm{RIoU}}$ and $L_{\mathrm{GD}}$. This configuration achieves the best overall performance with 70.1 AP50 and 35.9 mAP, outperforming all ablated variants. The progressive improvement from baseline to full model validates the design rationale of each component and demonstrates their effective synergy in addressing the challenges of SAR image rotated object detection.

### 5.5. Model Complexity and Inference Speed

We analyze model complexity and inference speed to evaluate the tradeoff between performance gains and computational cost. Table 7 reports parameter counts and frames per second (FPS) on an NVIDIA RTX 5880 Ada GPU with 800 × 800 input. This analysis examines the relationship between model complexity and detection performance.

The baseline Rotated-FCOS achieves 37.2 FPS with 32.1M parameters. Adding the KAN predictor (+KAN) introduces almost no additional parameters (32.2M vs. 32.1M). The FPS drops slightly to 35.4 due to the increased computational cost of KAN’s learnable spline functions. By contrast, adding wavelet features increases the parameters to 57.0M, and FPS drops to 27.3 due to the newly added wavelet backbone branch. Our full WE-KAN model combines both components. It has 57.1M parameters and runs at 26.3 FPS. This configuration introduces a significant increase in model capacity, leading to a noticeable drop in inference speed. However, it achieves a substantial 3.4-point gain in AP50 over the baseline. This demonstrates that the tradeoff between speed and accuracy can be justified when detection performance is the primary concern.

We also provide a comparison with other detectors. Among them, two-stage methods are significantly slower; for example, RoI-Transformer and O-RCNN run at only 13.5 FPS and 8.2 FPS. Transformer-based detectors also suffer from low efficiency, with Deformable DETR achieving 13.1 FPS with 41.0M parameters. Among one-stage methods, S^2^A-Net reaches 29.2 FPS with 36.4M parameters, while R^3^Det achieves 31.3 FPS with 41.9M parameters. These comparisons show that our method achieves competitive speed with one-stage detectors while delivering superior accuracy.

Moreover, we further investigate the performance of our method with the ResNet18 backbone for real-time applications. It uses only 30.4M parameters and runs at 35.3 FPS, nearly matching the baseline speed. Despite the reduced backbone capacity, it achieves 68.3 AP50, outperforming many existing methods including S^2^A-Net (66.5) and RoI-Transformer (66.9). This demonstrates that our core contributions remain effective even with lighter backbones. The ResNet18 variant provides a suitable option when deployment efficiency is critical.

In summary, WE-KAN introduces additional complexity, primarily through the wavelet feature branch. The KAN predictor itself introduces negligible parameter increase, with a slight impact on inference time. The consistent gains observed across different configurations confirm that the additional complexity translates into meaningful performance improvements. When speed is the priority, it is also possible to balance accuracy and efficiency by choosing lightweight backbones.

### 5.6. Visualization Analysis

**Figure 6:**
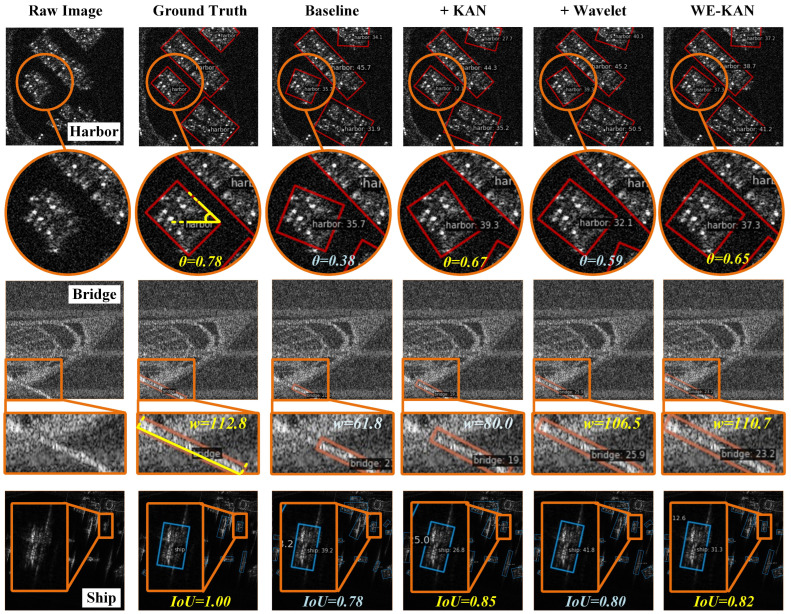
Visualization of rotated object detection results in WE-KAN ablation study: (row1) ground truth and predicted angles; (row2) width prediction for slender objects; (row3) IoU scores between predictions and ground truth.

The ablation studies in Section 5.4 quantitatively demonstrate the contribution of each component. To complement these findings with qualitative evidence, we perform visualization analysis using models from the ablation study. Figure 6 shows the prediction results on test set samples, comparing predicted angles, target lengths, and IoU scores across different model variants.

In Figure 6, the effects of different components can be intuitively observed through quantitative comparisons. The first row visualizes angle prediction performance for a harbor scene. The ground truth rotation angle is 0.78. The baseline method predicts 0.38, showing a substantial deviation. Introducing the KAN predictor alone improves the prediction to 0.67, while adding wavelet features alone yields 0.59. Our full WE-KAN model achieves 0.65, demonstrating that the KAN predictor contributes most significantly to angle regression accuracy.

The second row examines width prediction for a slender bridge object, where structural integrity is critical. The ground truth bridge length is 112.8 pixels. The baseline method predicts only 61.8 pixels, failing to capture the complete structure due to speckle noise and blurring. Adding the KAN predictor improves the prediction to 80.0 pixels, while introducing wavelet features alone achieves 106.5 pixels, substantially closer to the ground truth. The full WE-KAN model further refines this to 110.7 pixels. This clearly demonstrates that wavelet features are essential for structural perception and noise suppression, enabling accurate delineation of slender objects.

The third row compares IoU scores between predicted and ground truth bounding boxes. The baseline method achieves an IoU of 0.78. Adding the KAN predictor increases this to 0.85, while adding wavelet features alone reaches 0.80. The full WE-KAN model attains 0.82. The superior performance of the KAN predictor variant aligns with its highest AP75 score in Table 6. This confirms that precise angle regression directly translates to improved overall detection quality. Together, these visualizations provide intuitive evidence. Wavelet features enhance structural perception, while the KAN predictor excels at accurate angle regression. Their combination in WE-KAN delivers balanced and robust performance.

Overall, each component in WE-KAN contributes positively to the final performance. Specifically, the KAN predictor leverages its learnable spline activation functions to accurately capture the nonlinear relationships involved in angle regression. This capability leads to significantly improved angular precision. The wavelet domain module addresses the degradation in SAR images due to noise and low contrast, effectively converting vague grayscale details into enhanced feature representations. The designed modules work synergistically and effectively, collectively leading to superior prediction performance.

**Figure 7:**
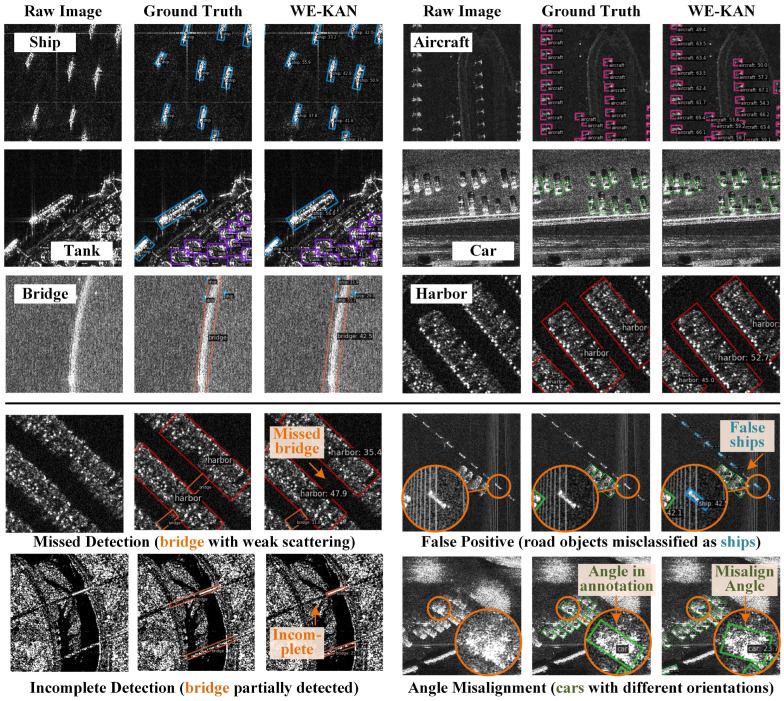
Representative detection results of WE-KAN on the RSAR dataset. Upper: successful detections across six categories including ships, aircraft, tanks, cars, bridges, and harbors. Lower: typical failure cases covering detection issues (missed detection, false positive) and regression issues (incomplete detection, angle misalignment).

To further demonstrate the effectiveness and limitations of our method, we provide additional visualization results in Figure 7. The figure is organized into two parts: successful detections in the upper part, and representative failure cases in the lower part.

As shown in the upper part of Figure 7, our WE-KAN achieves successful detection across all six categories in the RSAR dataset: ships, aircraft, tanks, cars, bridges, and harbors. For small and densely packed objects such as tanks and cars, the method maintains robust detection performance even under heavy clutter and limited pixel resolution. For large-scale structures such as harbors and bridges, WE-KAN produces tightly fitted bounding boxes with accurate orientation angles. These results demonstrate strong generalization across diverse target types and scales.

The lower part of Figure 7 illustrates four typical failure cases. The first two are detection-related issues. A weak-scattering bridge is completely missed, while road markings are falsely detected as ships due to their similar appearance. The remaining two are regression-related issues. A bridge with inhomogeneous scattering is only partially detected, and cars exhibit angle inaccuracies where surrounding parallel parking context is not effectively leveraged. These cases highlight remaining challenges in SAR rotated object detection, including handling low-contrast targets, suppressing structured background clutter, and incorporating contextual cues for orientation refinement. Addressing these issues will guide our future work.

### 5.7. Limitations and Future Work

Despite the promising performance of WE-KAN, several limitations should be acknowledged. First, WE-KAN introduces higher computational cost compared to other detection methods. As shown in Table 7, the full WE-KAN model contains 57.1M parameters, nearly double the baseline Rotated-FCOS (32.1M). This leads to a drop in inference speed from 37.2 FPS to 26.3 FPS. The wavelet feature extraction branch is the primary source of this increase, with the KAN predictor adding only minimal parameters (32.2M vs. 32.1M). Due to the fundamental differences between wavelet features and intensity features, we design a separate backbone for wavelet extraction. This design contributes to improved detection performance; however, it also introduces a tradeoff that may limit deployment in resource-constrained or real-time scenarios.

Second, generalization across SAR domains requires further validation. Although WE-KAN performs well on RSAR and RSDD-SAR, SAR imagery varies widely in imaging modes, resolutions, and geographical regions. The wavelet scattering coefficients are configured with fixed parameters (J=1, L=8) optimized on RSAR, and may not transfer optimally to datasets with different noise characteristics. Thus, more extensive evaluation on diverse SAR benchmarks would be a valuable direction for further exploration.

Third, certain challenging targets remain difficult for our method. As shown in the failure cases in Figure 7, objects in low-contrast regions or those with similar appearances may be missed or falsely detected. In terms of regression, targets with inhomogeneous scattering present another challenge, often being only partially detected or suffering from inaccurate angle prediction. These issues suggest that the current wavelet and attention mechanisms may not fully recover signals against noisy backgrounds. Developing more effective model architectures that leverage contextual semantic cues would be a valuable direction for future research.

Future work will focus on addressing these limitations through three main directions: first, lightweight wavelet feature extraction, such as employing knowledge distillation or designing more efficient backbone architectures, will be explored to reduce computational cost; second, adaptive wavelet configurations will be investigated to improve cross-dataset generalization, enabling the model to better handle varying noise characteristics across different SAR imaging modes; third, spatial context and prior knowledge will be incorporated to further improve detection accuracy in complex scenes, particularly for challenging targets with low contrast or inhomogeneous scattering.

Beyond remote sensing, the proposed framework holds potential for broader applications. It may provide benefits in other domains where object orientation and background clutter present similar challenges [12,13]. Adapting our wavelet domain feature enhancement and KAN-based angle prediction to these areas could improve detection robustness. Exploring such cross-domain applications will be another important future direction.

## 6. Conclusions

This paper addresses key challenges in SAR image rotated object detection, including speckle noise interference, varying object orientations, and complex backgrounds.

To address these issues, we propose a rotated object detection framework named WE-KAN. WE-KAN incorporates three specific designs: a wavelet-domain feature enhancement module, a KAN-based angle prediction head, and a joint loss function. The proposed method significantly improves the accuracy and robustness of rotated object detection.

First, a wavelet domain feature enhancement mechanism is constructed. It performs dual-branch feature extraction and attention-based fusion of SAR images with their wavelet scattering coefficients. Second, the Kolmogorov–Arnold network is introduced into the rotated object detection task, leveraging its learnable spline functions to enhance angle prediction accuracy. Finally, a joint loss function is designed that integrates rotated IoU loss and Gaussian distance loss to increase sensitivity to object shape.

Multiple experiments on the public RSAR dataset show that WE-KAN achieves an AP50 of 70.1 and an mAP of 35.9. The proposed method demonstrates significant advantages in slender targets and objects against complex backgrounds. Ablation studies and parameter analyses further validate the effectiveness and rationality of each module. Together, they conclusively demonstrate the efficacy of the proposed method in advancing SAR rotated object detection. The complete code and pretrained models for WE-KAN are publicly available at https://github.com/neulmc/WE-KAN (accessed on 26 February 2026) to ensure full reproducibility.

## Figures and Tables

**Table 1:** Performance metrics (AP50, AP75, and mAP) for different rotated object detection methods on the RSAR dataset.

Method	Backbone	Schedule	AP50	AP75	mAP
Rotated-RetinaNet [48]	ResNet50	1×	57.7	22.7	27.7
R^3^Det [20]	ResNet50	1×	63.9	25.0	30.5
S^2^ANet [21]	ResNet50	1×	66.5	28.5	33.1
Rotated-Faster RCNN [15]	ResNet50	1×	63.2	24.9	30.5
O-RCNN [19]	ResNet50	1×	64.8	32.7	33.6
ReDet [34]	ReResNet50	1×	64.7	32.8	34.3
RoI-Transformer [45]	ResNet50	1×	66.9	32.7	35.0
Deformable DETR [61]	ResNet50	3×	46.6	13.1	19.6
ARS-DETR [33]	ResNet50	3×	61.1	29.0	31.6
Rotated-FCOS [47]	ResNet50	1×	66.7	31.5	34.2
WE-KAN (Ours-R50)	ResNet50	1×	70.1	32.5	35.9
WE-KAN (Ours-R101)	ResNet101	3×	74.6	32.8	37.5

**Table 2:** AP50 scores for the six categories of different rotated object detection methods.

Method	Aircraft	Bridge	Car	Harbor	Ship	Tank	AP50
Rotated-RetinaNet [48]	73.5	49.6	73.6	53.4	73.6	22.4	57.7
R^3^Det [20]	73.2	56.9	89.3	63.0	78.7	22.6	63.9
S^2^ANet [21]	77.8	60.2	89.8	63.0	82.3	25.8	66.5
Rotated-Faster RCNN [15]	76.8	54.7	89.5	49.0	78.3	30.8	63.2
O-RCNN [19]	75.3	56.2	89.7	58.5	79.4	29.7	64.8
ReDet [34]	78.1	55.0	89.5	61.1	79.0	25.6	64.7
RoI-Transformer [45]	76.5	57.4	90.1	64.4	85.9	27.5	66.9
Deformable DETR [61]	51.3	36.8	66.5	45.4	58.0	21.7	46.6
ARS-DETR [33]	70.2	51.2	80.4	59.1	76.9	29.1	61.1
Rotated-FCOS [47]	73.0	58.8	89.8	65.5	79.0	33.9	66.7
WE-KAN (Ours-R50)	74.1	62.1	90.0	74.3	79.2	40.8	70.1
WE-KAN (Ours-R101)	70.3	64.8	89.9	77.6	86.5	58.1	74.6

**Table 3:** AP50 results on the RSDD-SAR dataset for inshore (InS) scenes, offshore (OffS) scenes, and all scenes.

Method	Backbone	Schedule	InS AP50	OffS AP50	AP50
Rotated-RetinaNet [48]	ResNet50	1×	33.2	74.1	66.7
R^3^Det [20]	ResNet50	1×	56.9	90.2	80.9
S^2^ANet [21]	ResNet50	1×	63.3	93.1	87.9
Rotated-Faster RCNN [15]	ResNet50	1×	48.8	90.9	83.3
O-RCNN [19]	ResNet50	1×	65.9	90.2	88.8
ReDet [34]	ReResNet50	1×	61.9	90.3	88.4
RoI-Transformer [45]	ResNet50	1×	60.8	94.4	88.4
CFA [62]	ResNet50	1×	66.4	90.5	89.3
Rotated-FCOS [47]	ResNet50	1×	50.0	93.1	85.5
WE-KAN	ResNet50	1×	66.7	90.3	89.4

**Table 4:** Different balance coefficients corresponding to the weights of the two losses and their associated AP scores on the RSAR dataset.

τ	$L_{\mathrm{RIoU}}$	$L_{\mathrm{GD}}$	AP50	AP75	mAP
0.1	0.1	0.9	69.5	31.6	35.3
0.2	0.2	0.8	69.1	32.5	35.4
0.3	0.3	0.7	69.2	31.9	35.3
0.4	0.4	0.6	69.9	32.3	35.5
0.5	0.5	0.5	70.1	32.5	35.9
0.6	0.6	0.4	69.9	32.8	35.6
0.7	0.7	0.3	70.0	31.9	34.9
0.8	0.8	0.2	69.8	32.3	35.3
0.9	0.9	0.1	69.3	32.4	35.2

**Table 5:** Sensitivity analysis of wavelet configuration parameters on the RSAR dataset.

Decomposition Level (*J*)	Directions (*L*)	Channels	AP50	mAP
1	4	5	68.9	35.0
1	8	9	70.1	35.9
1	16	17	69.7	35.3
2	4	25	69.0	35.1
2	8	81	70.2	35.8
2	16	289	69.6	35.1

**Table 6:** Ablation experiments on the proposed method in terms of input, predictor, and loss functions for the RSAR dataset.

Model	Input	Predictor	Loss	AP50	AP75	mAP
Baseline	Image	MLP	$L_{\mathrm{RIoU}}$	66.7	31.5	34.2
Baseline (GD loss)	Image	MLP	$L_{\mathrm{GD}}$	67.6	29.4	33.9
+Fusion Loss	Image	MLP	$L_{\mathrm{RIoU}}$, $L_{\mathrm{GD}}$	68.7	32.0	34.7
+Wavelet	Image & Wavelet	MLP	$L_{\mathrm{RIoU}}$	69.0	31.8	35.0
+KAN	Image	KAN	$L_{\mathrm{RIoU}}$	68.1	32.7	35.1
+Wavelet&KAN	Image & Wavelet	KAN	$L_{\mathrm{RIoU}}$	68.9	32.5	35.5
+Wavelet&KAN	Image & Wavelet	KAN	$L_{\mathrm{GD}}$	69.3	32.0	35.0
WE-KAN (Full)	Image & Wavelet	KAN	$L_{\mathrm{RIoU}}$, $L_{\mathrm{GD}}$	70.1	32.5	35.9

**Table 7:** Comparison of model complexity, inference speed, and detection performance across different method categories on the RSAR dataset.

Type	Method	Backbone	Params (M)	FPS	AP50
Two-Stage	Rotated-Faster R-CNN	ResNet50	41.3	14.8	63.2
	O-RCNN	ResNet50	41.3	8.2	64.8
	ReDet	ReResNet50	31.6	13.8	64.7
	RoI-Transformer	ResNet50	55.3	13.5	66.9
DETR-based	Deformable DETR	ResNet50	41.0	13.1	46.6
	ARS-DETR	ResNet50	41.4	12.9	61.1
One-Stage	R^3^Det	ResNet50	41.9	31.3	63.9
	S^2^ANet	ResNet50	36.4	29.2	66.5
	Baseline (Rotated-FCOS)	ResNet50	32.1	37.2	66.7
One-Stage (Ours)	+KAN	ResNet50	32.2	35.4	68.1
	+Wavelet	ResNet50	57.0	27.3	69.0
	WE-KAN	ResNet18	30.4	35.3	68.3
	WE-KAN	ResNet50	57.1	26.3	70.1
	WE-KAN	ResNet101	95.1	23.1	74.6

## Data Availability

The source code and pretrained model analyzed in this study are publicly available and can be accessed at: https://github.com/neulmc/WE-KAN (accessed on 26 February 2026).
